# Recent advances in synthetic biology of cyanobacteria for improved chemicals production

**DOI:** 10.1080/21655979.2020.1837458

**Published:** 2020-10-30

**Authors:** Fen Wang, Yuanyuan Gao, Guang Yang

**Affiliations:** a Department of Surgery, College of Medicine, University of Florida, Gainesville, FL, USA; b Jining Academy of Agricultural Science, Jining, Shandong, China; c Department of Aging and Geriatric Research, Institute on Aging, University of Florida, Gainesville, FL, USA

**Keywords:** Cyanobacteria, synthetic biology, promoters, ribosome binding sites, Crispr-cas9, riboswitches, chemical production

## Abstract

Cyanobacteria are Gram-negative photoautotrophic prokaryotes and have shown great importance to the Earth’s ecology. Based on their capability in oxygenic photosynthesis and genetic merits, they can be engineered as microbial chassis for direct conversion of carbon dioxide to value-added biofuels and chemicals. In the last decades, attempts have given to the application of synthetic biology tools and approaches in the development of cyanobacterial cell factories. Despite the successful proof-of-principle studies, large-scale application is still a technical challenge due to low yields of bioproducts. Therefore, recent efforts are underway to characterize and develop genetic regulatory parts and strategies for the synthetic biology applications in cyanobacteria. In this review, we present the recent advancements and application in cyanobacterial synthetic biology toolboxes. We also discuss the limitations and future perspectives for using such novel tools in cyanobacterial biotechnology.

## Graphical abstract

**Figure:**
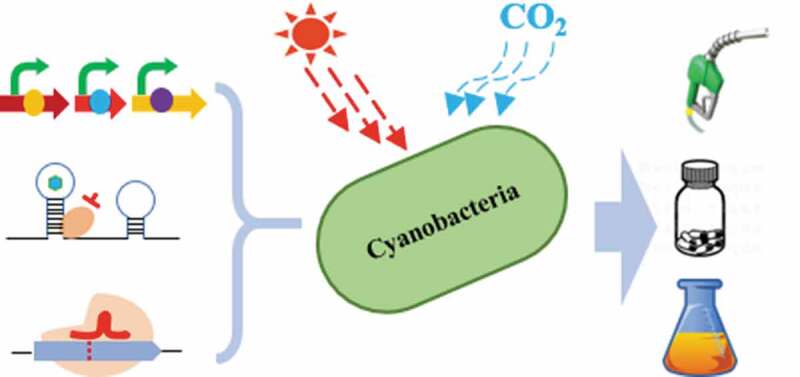

## 1. Introduction

Cyanobacteria are a group of diverse and ubiquitous Gram-negative photoautotrophic prokaryotes. They can perform oxygenic photosynthesis using sunlight as energy source to transform carbon dioxide into biomass. On the one hand, cyanobacteria have been contributing to the rise of oxygen in Earth’s atmosphere since a billion years ago [1]. On the other hand, they also play essential roles in carbon and nitrogen cycling in the oligotrophic oxygen-deficient environments [2]. In comparison with other photosynthetic organisms (e.g., plants and algae), cyanobacteria are often fast-growing with higher production rates [3,4]. Moreover, the amenability to genetic manipulations and high metabolic plasticity make some of the cyanobacteria species attractive targets for photosynthesis studies and potential hosts for light-powered biotechnological applications.

Over the last decades, cyanobacteria have been successfully engineered as chassis for the production of a variety of valuable chemicals, such as fatty acids [5], ethylene [6], ethanol [7], 1-butanol [8], sucrose [9], shinorine [10], terpenoids [11], etc. However, the yield of the products in most of the cases have been low compared to the reported counterparts from the more conventional hosts, such as *Escherichia coli* and yeast. One of the most important reasons for such a low productivity with cyanobacteria is the lack of fine-tunable genetic regulatory elements and enabling technologies, which are major limitations to achieve their full potential.

The crux of synthetic biology promotes a bottom-up approach to redesign biological platforms with recombination of defined parts, modules or artificial regulatory circuits [12]. In the past, synthetic biology strategies have been used in the development of both *E. coli* and *Saccharomyces cerevisiae* as prokaryotic and eukaryotic production hosts, resulting in tremendous successes [13,14]. Similar approaches may also be used in cyanobacteria to refine photosynthetic yield and carbon flux toward the product of interest, thus unlocking the full power of cyanobacteria for microbial cell factories [15]. The therefore engineered cyanobacterial hosts possessing modulated metabolisms will facilitate generation of desired outputs such as chemicals and proteins [16].

Although efforts have given to the development of cyanobacterial cell factories for decades [17], the use of synthetic biology strategies in such applications is still in its infancy [18]. To bridge the gap between the requirements for enhanced titers of the products that are aimed toward commercial applications and limited number of advanced cyanobacteria synthetic biology tools, studies have recently focused on the following three aspects: 1) characterizing native and synthetic promoters and ribosome binding sites (RBS); 2) using riboswitches, selectable markers, suitable vectors for stable chromosome integration and dynamic regulation of gene expression; and 3) using genome-wide editing and regulating tools.

In this review, we have summarized the recent developments and applications in cyanobacterial synthetic biology toolboxes. In addition, we also carefully discussed the limitations and potential future directions for using these new tools in cyanobacterial biotechnology. This review aims to provide not only the state-of-the-art advancements but also insights into the current challenges and future perspectives in development of cyanobacterial synthetic biology strategies and engineering novel cyanobacterial genetic tools.

## 2. Advances in synthetic biology toolboxes for cyanobacteria

### 2.1. Promoters

Promoters are key synthetic biology tools that have been studied for diverse cyanobacterial species. In general, the commonly characterized promoters can be classified into two groups according to their functional differences, including inducible and constitutive promoters. A list of recently characterized promoters are summarized in Table 1, for the ease of reference.

**Table 1.** List of recently characterized promoters for cyanobacteria

Name	Tested host	Description	Reference
**Inducible**			
PL03	*Synechocystis* 6803, *Anabaena* 7120	Anhydrotetracycline-inducible promoter. The induction range was 200-fold in *Synechocystis* 6803. When tested in *Anabaena* 7120, the PL03-driving expression of GFP reached 7% of the total protein.	[19,29]
Psca6-2	*Synechocystis* 6803	A variant based on *E. coli* Ptac with approximately 10-fold induction ratios.	[20]
PO2	*Synechocystis* 6803	Dark or anaerobic activation promoter.	[44]
PEZtet	*Synechococcus* 7002	Hybrid of PcpcB and two tet operators with a 32-fold dynamic range.	[33]
PcptOO-cLac143	*Synechococcus* 7002	IPTG-inducible promoter with 48-fold dynamic range,	[34]
PompC	*Synechocystis* 6803	Dark activation promoter.	[42]
Pvan	*Synechococcus* 7942	Vanillate-inducible promoter.	[39]
PBAD	*Synechocystis* 6803, *Synechococcus* 7942	L-arabinose activation promoter.	[35,42]
PrhaBAD	*Synechocystis* 6803	Rhamnose-activation promoter.	[37]
PvanCC	*Synechocystis* 6803	Vanillate-inducible promoter with a 16-fold dynamic range.	[38]
**Constitutive**			
PpsbA	*Synechocystis* 6803, *Synechococcus* 7942	The activity of limonene synthase was enhanced by 100-fold under PpsbA than that under Ptrc in *Synechococcus* 7942.	[6,21]
Pcpc560	*Synechocystis* 6803	Pcpc560-driving expession of proteins results in about 15% of the total soluble proteins.	[56]
PR-PS	*Synechococcus* 7942	Proteins generating by PR-PScould account for about 12% of the total extracted proteins.	[22]
Ptrc	*Synechocystis* 6803, *Synechococcus* 2973	A *E. coli-*derived promoter that was used to drive the expression of yfp.	[23,61]
Psca3-2	*Synechocystis* 6803	A Ptac-variant promoter with modarate activity.	[20]
Plac	*Synechococcus* 2973	A *E. coli-*derived promoter that was used to drive the expression of *cscB* for sucrose production.	[9]
PpsbA2S	*Synechocystis* 6803	A derivative of PpsbA2 promoter with shorter sequence. It shows 4-fold higher strength when compared to its original version.	[67]
PA2520	*Synechococcus* 7002	PA2520 showed about 8-fold higher strength than Prbc of *Synechococcus* 7002.	[57]
PA2579	*Synechococcus* 7002	PA2579 showed about 8-fold higher strength than Prbc of *Synechococcus* 7002.	[57]

#### 2.1.1. Inducible promoters

The development of synthetic biology hosts often involves introduction of genetic pathways that exert heavy metabolic loads or having generated metabolites harmful to the cells [24]. In such cases, inducible promoters are especially crucial to ensure the successful application of genetic modifications and, consequently, the development of new cultivars resistant to stress. In early studies, the most standard inducible promoter systems used in cyanobacteria are derived from *E. coli*. For example, the isopropy-*β*-D-thiogalactoside (IPTG)-induced Ptrc promoter and its variants are based on the lac-operon in *E. coli* [25,26]. These promoters were characterized as poorly responsible in different cyanobacterial species, which limits their applications [27–29]. In contrast, the L03 promoter, derived from *E. coli*-derived induction system of the tetracycline-resistance operon TN10, induced by anhydrotetracycline (aTc) were able to function in several cyanobacterial hosts [29], including *Synechococcus elongatus* pcc 7942 (hereafter *Synechococcus* 7942), *Synechococcus* sp. PCC 7002 (hereafter *Synechococcus* 7002) and *Synechocystis* sp. PCC 6803 (hereafter *Synechocystis* 6803) [30–32]. Despite its wide applicability in cyanobacterial synthetic biology, the inherent light-degradation property of aTc makes this kind of promoters difficult to control in the photoautotrophic hosts, particularly when stable and sustained induction is desired [33]. Recently, attempts were given to the combination of aTc- or IPTG-based induction systems with strong constitutive promoters for the development of inducible promoters with high strength. Several promoters were thus generated showing moderate expression and tight control of induction, including PEZtet with combination of the PcpcB and two tet operators [33] and PcptOO-cLac143 containing Pcpt-lac operator hybrids [34].

A third set of inducible promoters, including PBAD, PrhaBAD and Pvan, are based on the xylose-metabolic pathway or the glucose-tolerance properties upon the mixotrophic cultivation of cyanobacteria. The arabinose-inducible PBAD promoter was first characterized in *Synechococcus* 7942 with a relatively high strength and can be well-repressed in the absence of the inducer [35,36]. Similar findings were observed when the rhamnose-induced and RhaS-regulated promoter PrhaBAD was used in *Synechocystis* 6803, where a moderate activity and tight repression were observed [37]. This promoter was thus recognized as one of the most robust inducible promoter systems for the applications in *Synechocystis* 6803 [38]. The Pvan promoter is suppressed and induced by *Corynebacterium glutamicum* VanR and vanillate, respectively. Previously, this promoter was characterized to be only functional in *Synechococcus* 7942 with tight control but low strength, resulting in a 50-fold dynamic range [39]. Very recently, the vanR/PvanCC promoter system from *Caulobacter crescentus* was further optimized for its application in *Synechocystis* 6803, showing a tightly controlled linear dose-response to vanillate with a 16-fold dynamic range [38].

Given the essential role of photosynthesis in the metabolism of cyanobacteria, it is reasonable that the light/dark-induced and O_2_-dependent promoters are also applied in heterologous expression systems in cyanobacteria. Previously, the most recognized light-responsive promoters are the PpsbA derived from plant *Amaranthus hybridus* [40] and its variants [41]. Recently, Immethun and colleagues have reported a novel darkness-induced promoter system consists of the hybrids of native light sensor protein Cph1 from *Synechocystis* 6803 and the kinase EnvZ from *E. coli* [42]. In response to the darkness, Cph1 phosphorylates its linked histidine kinase domain (EnvZ). The latter then further phosphorylates the *E. coli*-derived transcription factor OmpR, leading to activation of the promoter PompC. Although a low productivity has been reported for this system, the promoter could be particularly useful in the control of processes that require darkness, such as the butanol production in *Synechococcus* 7942 [42,43]. Meanwhile, the same research group has developed an oxygen-responsive promoter PO_2_ that can be induced by the FNR (fumarate nitrate reductase) under anaerobic conditions in the dark [44]. This promoter was applied in *Synechocystis* 6803 resulting in a moderate expression level of the flavin-binding fluorescent protein (FbFP), under low O_2_ conditions [44].

Besides the above-discussed promoters that are originated from heterologous elements, potential inducible promoters could also be obtained from the native cyanobacterial genomes. Indeed, a number of native promoters that have previously been characterized to be responsible for heavy metals, light, salt, nutrition starvation and so on are already applied successfully in various cyanobacterial research and biotechnology applications. In terms of the fact that over 400 cyanobacterial genomes are available in public databases to date [45], together with the large amount of cyanobacterial transcriptomic and proteomic data under diverse stresses [46–53], provide tremendous potential for screening of inducible promoters.

#### 2.1.2. Constitutive promoters

Constitutive promoters are used to drive stable and continuous expression of target genes or to fine-tune synthetic biology pathways where the regulated expression is not necessary. A collection of the most commonly used constitutive promoters are discovered natively from cyanobacteria, such as PcpcB and PpsbA2. These promoters play essential roles in driving the expression of key components in the photosynthetic pathways and thus are highly efficient and present in most if not all cyanobacterial species [54,55]. Recently, variants of PcpcB promoter, including Pcpc560 and Pcpt, were characterized and used in *Synechocystis* 6803 and *Synechococcus* 7002, showing moderate to high product yields [10,33,34,56].

Another inherent promoter commonly used in cyanobacterial host development is the RuBisCO promoter PrbcL [57]. Its variants were characterized from several cyanobacterial species, such as *Anabaena* sp. PCC 7120 (hereafter *Anabaena* 7120) [40], *Synechocystis* 6803 [26] and *Synechococcus* 7942 [58]. Very recently, a group of inducer-free promoters has been generated through error-prone PCR of PrbcL and PcpcB, resulting in 48 novel promoters with a dynamic range of 2 orders of magnitude [Sen^59^]. Most importantly, these engineered promoters have shown diverse activities when tested in three cyanobacterial strains, thus expands the potential of using these cyanobacteria in the synthetic biology applications.

In some applications, continuous but weak gene expression may be required, where the constitutive promoters with low activity will be particularly useful. One example is the well-characterized constitutive promoter PrnpB from *Synechocystis* 6803, which initiates expression of the gene encoding the RNA subunit of ribonuclease P. Due to the low strength of this promoter, it may not be appreciable for overexpression of enzymes in biosynthetic pathways, however, can be used for expression of repressors that sometimes required for trace activity in regulatory circuits [27].

Although some of the promoters used for driving consistent expression of genes, they may not be truly constitutive due to their inducible activities under certain conditions. For example, the PpsbA is actually a light-inducible promoter, but has been commonly used in constitutive expression pathways under constant light conditions [60]. On the other hand, many previous mentioned inducible promoters can also support constitutive expression in the absence of their specific regulators. The orthogonal promoters such as Ptrc or Ptrc2O have been successfully used for high yield expression of proteins in constitutive expression systems as they are highly efficient in the absence of repressor [26,32,61].

### 2.2. Ribosome binding sites

Apart from the promoter, RBS can also determine the level of gene expression by mediating the rate of ribosome recruitment for translation. It has long been recognized that the sequence and position of given RBS significantly affect translational efficiency [62,63]. Previously, methods were developed for predicting and controlling translation initiation and protein expression in *E. coli* [64–66]. Until recently, such effort has not been extended toward development of RBS libraries for the control of gene expression in cyanobacteria. Early studies reported characterization of BioBrick registered RBSs in *Synechocystis* 6803 [67,68]. Another recent study has investigated 20 native RBS elements in the same cyanobacterial strain [69]. Early application of RBS regulation of translational efficiency was in *Synechococcus* 7942, where four *E. coli* RBSs with different dynamics were used to mediate expression of heterogeneous pathway genes for production of 2,3-butanediol, resulting in a significant enhancement of yield of product [70]. With recently increased number of researches in the construction of cyanobacterial RBS libraries, it can be anticipated that the use of RBS will soon become another important appliance to expand the cyanobacterial synthetic biology toolbox.

### 2.3. Riboswitches

Riboswitches are cis-activating or cis-repressing regulatory elements most likely present in the 5ʹ untranslated regions of mRNAs [71,72]. They usually impose a secondary-structural conformation on the mRNA to control translational efficiency of the transcript [73]. They have a widespread distribution of taxonomy and are capable of regulating highly conserved metabolic pathways, indicating their long history of gene regulatory mechanism [74]. To date, up to 50 classes of riboswitch are investigated, which involved in a variety of crucial biochemical pathways including co-enzymes, nucleobases, amino acids and single ions [74,75]. In cyanobacteria, only a few riboswitches are studied for their application in regulation of gene expression. Previously, a modified theophylline-dependent riboswitch was tested in *Synechococcus* 7942, resulting in a strictly controlled protein expression system with a maximum 190-fold of dynamic range [76]. Recently, this riboswitch has been employed in several other cyanobacteria stains, including *Synechocystis* 6803, *Leptolyngbya* sp. BL0902 (hereafter *Leptolyngbya* BL0902), *Anabeana* 7120 and *Synechocystis* sp. strain WHSyn (hereafter *Synechocystis* WHSyn) [77–79]. In another recent attempt, Taton et al. have used theophylline-dependent synthetic riboswitches to control transcriptional repressors, thus downregulating gene expression in five strains of cyanobacteria (i.e., *Anabeana* 7120, *Synechocystis* 6803, *Synechococcus* 7942, *Leptolyngbya* BL0902 and *Synechocystis* WHSyn) [39]. Most recently, a theophylline-responsive riboswitch has been hybridized with the rhamnose-induced PrhaBAD promoter to initiate the expression of a CRISPR interference (CRISPRi) mechanism that represses photosystem II activity and thus restrain the growth of *Synechocystis* 6803 under nutrient limitation conditions [80]. Importantly, this combined system is reversible by removing the inducers rhamnose and theophylline. This design may provide a new approach for applying combination of various regulatory mechanisms in finetuning expression of target genes.

### 2.4. CRISPR‐based genome editing and transcriptional regulation

**Figure 1.**
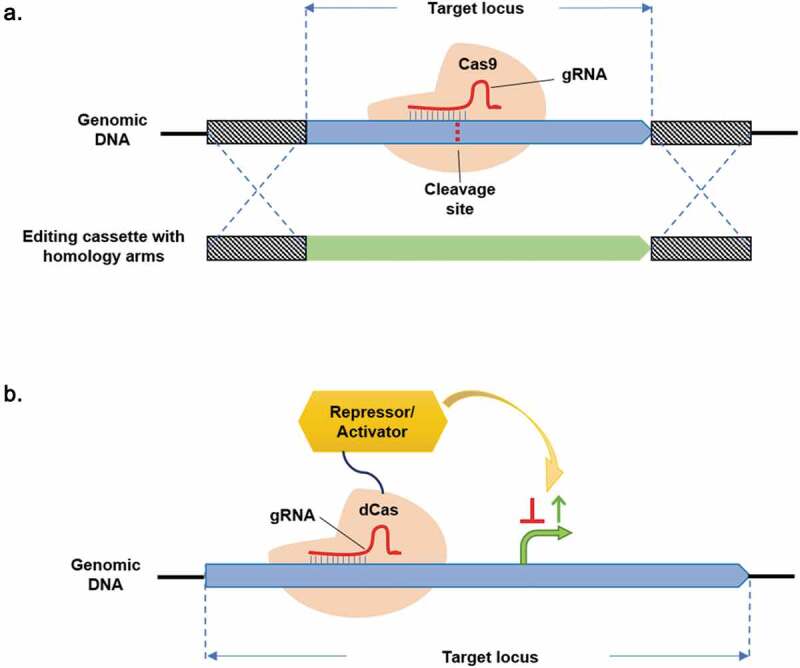
CRISPR-Cas systems for genome editing and CRISPRi in cyanobacteria. (a) Schematic representation of CRISPR-Cas9 mediated genome editing in cyanobacteria. A double-strand DNA cleavage will be created by Cas9 after bond to target gene locus. Then the homologous recombination event will subsequently take place to repair the DNA damage, thus facilitating genome editing; (b) Schematic representation of CRISPRi in cyanobacteria, where dCas9-repressor results in repression of gene expression and dCas9-activator leads to activation of gene expression

Conventionally, genome modification in cyanobacteria is entirely based on homologous recombination through natural transformation or conjugation of plasmids or linear DNA fragments into the cells. Although homology recombination has proven to be an efficient approach for generating gene deletion, insertion, and nucleotide substitution in cyanobacteria model strains, simultaneous large-scale genome editing is still challenging. In addition, many cyanobacteria are oligoploid or polyploid [81], to obtain homozygous mutant strains a segregation step is often required to ensure all chromosome copies carry the same targeted mutation, however, this procedure involves many rounds of antibiotics selection and can be time consuming. The CRISPR-Cas system is well-recognized for its application in genome editing and has been successfully used in cyanobacterial genome modifications [82]. This machinery can cause double-strand DNA cleavage in the cyanobacterial chromosome, a homologous recombination event will subsequently take place to repair the DNA damage, thus facilitating genome manipulation (Figure 1(a)). Most importantly, the high efficiency of CRISPR-Cas system can significantly improve the frequency of genome editing and accelerate the segregation process [83,84]. Previously, the CRISPR-Cas9 mediated markerless deletion strategy has been developed in *Synechococcus elongatus* UTEX 2973 (hereafter *Synechococcus* 2973) for repairing the mutant *nblA* gene (an essential gene for phycobilisome degradation) into wild-type *nblA* [84]. This study provided a proof of concept work for the introducing a CRISPR-Cas9 system in cyanobacteria with successful removal of the edited exconjugants. Latterly, the CRISPR-Cas9 genome editing approach was employed in *Synechococcus* 7942 for metabolic engineering application, where the carbon flux has been redirected from glycogen to succinate synthesis pathway for improvement of the product titer [83]. Despite a few numbers of initial successes, it was also revealed that the accumulation of Cas9 protein could cause toxicity to the cell, thus limits its applications in engineering cyanobacterial genome [84]. To overcome such a restriction, a new CRISPR-Cas12a system has been developed showing less toxicity when compared to Cas9 in cyanobacteria [82,85]. This alternative system was successfully used in several cyanobacteria strains, including *Synechococcus 2973, Anabeana* 7120 and *Synechocystis* 6803, which demonstrated determination of cell lethality and the markerless gene replacement strategy [86,87].

Besides applications in genome editing, CRISPR-Cas systems are also used for CRISPRi to regulate gene expression in cyanobacteria. This was achieved by using deactivated Cas proteins (dCas) that have disabled for cleavage of target genes but are capable of interrupting transcriptional process (Figure 1(b)) [88]. Recently, both CRISPR-dCas9 and -dCas12a systems have demonstrated successful applications in several model cyanobacteria, such as *Synechocystis* 6803 [32,89,90], *Synechococcus* 7942 [91], *Anabaena* 7120 [92,93] and *Synechococcus* 2973 [86]. In these applications, CRISPRi was successfully employed for the dynamic up/down regulation of the target genes in various synthetic pathways for improved productivity of biofuels (e.g., fatty acids and fatty alcohols) and other important metabolites (e.g., amino acids, succinate, lactate and pyruvate). It is well expected that such technologies (i.e., CRISPR-Cas-based genome editing and CRISPRi) could provide more opportunity in development of cyanobacterial synthetic biology platform for building efficient bio-solar cell factories.

### 2.5. Other genetic tools for cyanobacteria

Finding a suitable selectable marker is a prerequisite for the selection of genetically engineered strains. In the past, antibiotics have been the most commonly used selectable markers in cyanobacteria, including chloramphenicol [94], erythromycin [95], kanamycin [94], spectinomycin [96], neomycin [97], streptomycin [98], spectinomycin [99], zeocin [100] and gentamicin [99]. Recently, new selectable markers were established by breaking down specific insertion sites leading to readily observable phenotypes. For example, in a study on the development of counter-selection system for cyanobacteria, a genetic insertion at the *acsA* (acetyl-CoA ligase) locus led to a selectable phenotype that is tolerant to acrylate [101]. More recently, Wendt et al. also reported that breakdown of *nblA* (phycobilisome degradation protein-coding gene) resulted in an obvious non-bleaching phenotype of *Synechococcus* 2973 under nitrogen starvation and can be used as a selectable marker [84]. Another alternative approach was latterly investigated in *Synechococcus* 7002, where expression of a heterologous phosphite dehydrogenase encoding gene in the absence of its cognate phosphite transporter allowed growth of the cyanobacterium on phosphite, thereby enabling the selection of mutant strain [102].

Another important topic of synthetic biology is to efficiently deliver the DNA of interest into the host cell. Plasmid vectors are designed to carry the genetic materials and have been used in transformation of cyanobacteria. The currently available cyanobacteria-specific vectors can be categorized into two groups, i.e. integrative and replicative vectors [103]. The former group is mainly used to deliver DNA cargo at genetic loci of interest in the host genome, thus allowing gene knockout or knock-in. The knock-in of heterologous DNA material usually requires an appropriate neutral site that can be disrupted without affecting cellular viability or cause any distinguishable phenotype [104]. The recent development of SyneBrick integrative vectors facilitates integration at three neutral sites [31]. It also contains set of three inducible promoter systems. The replicative vectors, on the other hand, are mainly employed for transient expression of target genes. The most commonly used replicative vectors are previously derived from a broad-host vector RSF1010, including pSL1211, pPMQAK1 and pFC1 [15,17,26]. Recent attempts have given to the modification of RSF1010-based vectors for improved transmissibility, increased copy numbers and ease of cloning [105,106]. The optimized vectors may therefore serve as more efficient synthetic biology tools in the development of cyanobacteria as chassis for light‐driven biotechnology.

## 3. Applications of synthetic biology in cyanobacteria production of chemicals

Considering the aforementioned inherent merits of cyanobacteria, they are one of the promising candidates for the sustainable production of biofuels and high-value chemicals. Recent advances of synthetic biology tools and strategies have significantly improved photosynthetic efficiency in cyanobacterial cell factories by remodeling cyanobacterial metabolism and physiology, and thereby enhancing the titer of the desired products. Such approaches have been applied to improve productivity of chemicals from both cyanobacterial primary and secondary metabolic pathways. In one recent study, Gupta and colleagues introduced a strong light-inducible promoter, PrbcL2A, and a strong RBS sequence in *Synechococcus* 7002 for an overexpression of two Na^+^-dependent carbon transporters, SbtA and BicA, resulting 50% increase of glycogen production from this strain [107]. In cyanobacteria, glycogen is used as source for the production of many sugars and carbohydrates in response to osmotic stress [108]. However, the accumulation of those products can be harmful to the cell due to the lack of sugar transporters in cyanobacteria [109]. Therefore, synthetic biology was also applied to introduce sugar transporters facilitating the export of the hydrophilic metabolites, thus keeping a healthier cell factory and in the meantime, allowing extracellular accumulation of carbohydrates for further biotechnological applications [109,110]. Polyunsaturated fatty acids (PUFAs) have shown exert anti-inflammatory and cardioprotective activities in cardiovascular disease and several inflammatory diseases. They have latterly been produced in *Anabaena* 7120, *Synechococcus* 7002 and *Leptolyngbya* BL0902, by expressing Acyl-lipid desaturases and Vipp1 in these cyanobacteria [111]. In addition, terpenoids are the largest group of plant secondary metabolites, which have been engineered as primary metabolic pathway products in cyanobacteria strains. Several previous review articles have well summarized a number of successful efforts in development of cyanobacterial platforms for production of terpenoids [11,112,113]. Recently, attempts were further given to the optimization of yield of the heterologous production of terpenoids in the engineered cyanobacterial strains. For example, in order to overcome the low expression levels of the key enzymes in the β-phellandrene biosynthetic pathways in *Synechocystis* 6803, fusion constructs technologies were introduced to increase enzyme production and catalytic efficiency, and resulting in elevation of the productivity of β-phellandrene by up to 4 and 8-fold in separate studies [114,115].

## 4. Challenges and future perspectives

Despite the foregoing successful proof-of-principle studies, wide application of cyanobacterial cell factories is still a technical challenge due to low yields of bioproducts [116]. One important limitation could be the relatively small number of sophisticated genetic regulatory components for achieving specific and tunable control of introduced genes and pathways in cyanobacteria [117]. Although a wide variety of well-established regulatory parts have been developed in other heterotrophic counterparts (e.g., *E. coli* and *Saccharomyces cerevisiae*), they may not always transferrable to cyanobacteria, leading to the delay in their advancement as industrial hosts [12,26]. Many recent attempts have been given to the characterization of standardized modular parts in cyanobacteria. Nevertheless, characterization of such parts could be significantly advanced by the establishment of robust and modular expression libraries [118]. In addition, the expansion of the genomic, expression and mutation libraries may also offer new insights into complex physiology of cyanobacteria, therefore facilitating metabolic engineering of model strains. On the other hand, CRISPR-based genome editing and interference approaches have proven to be potent and efficient tools in the regulation of gene expression by targeting multiple loci in parallel. However, the toxicity and off-target effects of the CRISPR nucleases still largely restricting its application in cyanobacteria genome modification [82]. Such an issue has been partially addressed by controlling the expression of CRISPR nucleases after introducing into cyanobacteria. Considering the current achievements in development of CRISPR-Cas systems in cyanobacteria, an invaluable toolkit can be anticipated for strain engineering in the future.

## 5. Concluding remarks

Cyanobacteria synthetic biology toolkits have been advancing rapidly in the last decade, offering tremendous opportunities for engineering cyanobacteria as feasible photosynthetic chassis for the solar manufacturing of commodity chemicals. Although extraordinary progresses have been implemented for using such genetic tools in development of cyanobacteria toward photosynthetic microbial cell factories, more efforts are still required for the construction of novel genetic circuits for fine-tuned dynamic control. This review outlined the recently developed cyanobacteria synthetic biology tools and strategies with a discussion of the obstacles and solutions for better using such new techniques in further development of cyanobacteria toward photosynthetic microbial cell factories.

## Highlights

- Synthetic biology tools are developed advancing cyanobacterial biotechnology.
- Riboswitches are used for dynamic control of protein expression in cyanobacteria.
- Crispr-cas systems are applied for genome editing and transcriptional regulation.
- Synthetic biology strategies improved chemicals production in cyanobacteria.

## Declaration of the authors

All authors have reviewed the manuscript and have agreed to the order of the authorship.
